# Recent Progress in Piezoelectric-Triboelectric Effects Coupled Nanogenerators

**DOI:** 10.3390/nano13030385

**Published:** 2023-01-18

**Authors:** Yifei Wang, Xia Cao, Ning Wang

**Affiliations:** 1 Center for Green Innovation, School of Mathematics and Physics, University of Science and Technology Beijing, Beijing 100083, China; 2 Beijing Institute of Nanoenergy and Nanosystems, Chinese Academy of Sciences, Beijing 100083, China

**Keywords:** piezoelectric-triboelectric nanogenerator, coupled effect, structure integration, performance enhancement

## Abstract

Piezoelectric and triboelectric nanogenerators have been widely studied in the past years for their advantages of easy design/manufacturing, small size, and flexibility. Nanogenerators that are developed based on the coupled piezoelectric and triboelectric effects (PTCNG) can make full use of the mechanical energies and achieve both higher output and sensing performance. This review aims to cover the recent research progress of PTCNG by presenting in detail their key technologies in terms of operating principles, integration concept, and performance enhancement strategies, with a focus on their structural simplification and efficiency performance improvement. The latest applications of PTCNG in tactile sensors and energy-harvesting system are also illustrated. Finally, we discuss the main challenges and prospects for the future development of PTCNG, hoping that this work can provide a new insight into the development of all-in-one mechanical energy-scavenging and sensing devices.

## 1. Introduction

Currently, artificial intelligence technology and Internet of Things (IoTs) are playing important roles in industry and people’s lives, from managing inventories to improving personalized shopping experiences and assisting in data mining, even transforming how people do business today [1]. Intelligent electronic devices such as wearable devices [2,3,4,5], implantable devices [6,7,8], medical robots, and other products are showing explosive growth and helping people control and detect various aspects of the surrounding environment [9], human health, and energy harvesting [10,11,12]. However, because the composition of artificial intelligence and IoTs require numerous sensors with high performance, low power consumption, and especially, stable and sustainable operation, which is the key to realize the full power of IoTs, traditional electrochemical sensor power sources are facing challenges in this era due to their limited endurance and energy storage capacity [13,14,15].

As a kind of emerging energy-harvesting technologies, nanogenerators, such as triboelectric nanogenerators (TENG) and piezoelectric nanogenerators (PENG), are proposed for scavenging mechanical energies from our ambient environment and transforming them into electricity in a distributed manner [16]. TENGs are fabricated based on electrostatic inductive coupling and the triboelectric effect, while PENG is developed based on the piezoelectric effect and generating alternating currents from the polarization, both with advantages of a high output voltage, simple fabrication, lightweight, and small sizes [17,18,19,20].

Since Wang et al. proposed the concept of a hybrid nano-energy-harvesting device for collecting mechanical energy and solar energy in 2009, the strategy of hybridizing or coupling multi-effects together has been widely applied to the design of nanogenerators for collecting single or multiple types of energy [21,22,23]. Among the various all-in-one multi-energy-harvesting devices, the synergism between piezoelectric and triboelectric effects is especially attractive due to the easiness to integrate that comes from their similarity in both structure and operation principle, which means they can be integrated together with a simpler structure, higher efficiency, and other complementary characteristics.

Generally, PTCNG structures are divided into three types [24,25,26,27,28,29,30]. In the two-end structure, piezoelectric and triboelectric characteristics are provided by one material. Electrostatic charges are directly added to the material surface, increasing the potential difference between the two surfaces of the film. In the three-terminal structure, TENG and PENG share one electrode, which can increase the triboelectric charge on the PENG electrode. For the four-terminal structure, PENG and TENG work independently. In addition, the polarity of piezoelectric material can be flexibly modulated, which can improve the conversion efficiency of the generator [31]. Previous studies have proved that PENG has the best energy conversion efficiency in the resonant state, which can be mutually coordinated with TENG to activate the resonant mode and reduce the output phase difference to achieve effective hybridization of PENG and TENG [32,33]. At the same time, PENG and TENG have the characteristic of complementary frequency, and both have high stable output power and conversion efficiency under weak low-frequency external stimulation, and thus may achieve efficient and stable mechanical energy collection [28,34]. In addition, due to the wide selection of piezoelectric and triboelectric materials, the impedance matching between PENG and TENG is not difficult, which can reduce the additional energy loss [35]. This helps the two-part work in concert with easy integration, enhanced overall charge density, high flexibility, light weight, and small size. Besides, both the responsivity and specific detectivity of sensors that are developed on the coupled device can be enhanced due to the frequency complementarity and multi-signal division. Consequently, spatially resolved force detection may be realized by recording the different output voltage signals as a mapping figure in smart devices [36,37].

**Figure 1:**
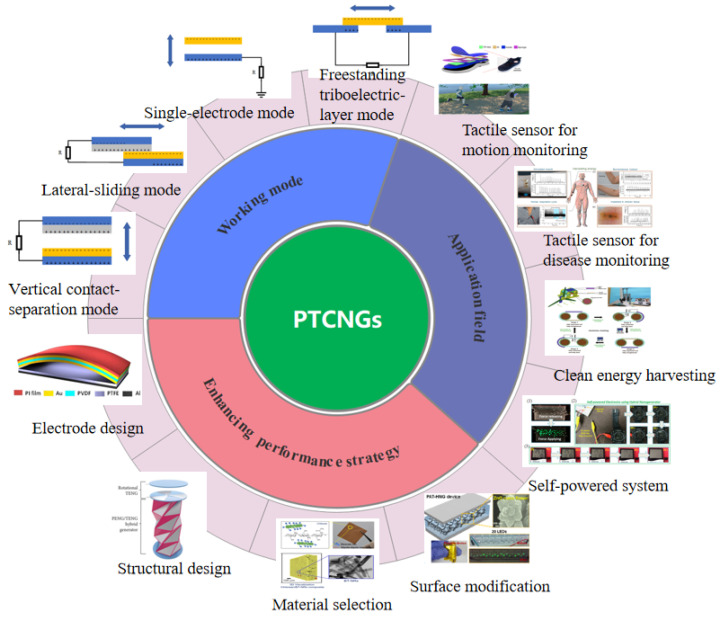
Recent progress of PTCNGs.

Here, we systematically review the recent research progress of PTCNG from the working principle (Figure 1). After illustrating the integration concept and performance enhancement strategies, the latest applications of PTCNG in tactile sensors and energy-harvesting systems are also introduced. Finally, the applications of PTCNG in tactile sensors and energy-harvesting systems are concisely covered, while highlighting the main challenges and prospects for the future development of PTCNG, in the direction of both structure optimization, materials’ selection, and multifunctional design. We believe that such integrated energy conversion devices may make greater contributions to our society.

## 2. The Working Principle of Piezoelectric/Triboelectric Nanogenerator

### 2.1. Piezoelectric Effect

The piezoelectric effect was discovered by Pierre Curie and others in the research of Rochelle salt, quartz, and other materials. Materials with an asymmetric crystal structure are piezoelectric materials, and common piezoelectric materials can be divided into two categories: inorganic piezoelectric materials and organic piezoelectric materials. Inorganic materials include piezoelectric ceramics and crystals, such as lead zirconate titanate (PZT), barium titanate (BaTiO_3_), lithium niobate (LiNbO_3_), zinc oxide (ZnO), and quartz. Meanwhile, representative organic piezoelectric materials are polyvinylidene fluoride (PVDF) fibers/films and their copolymers [38]. The piezoelectric effect of piezoelectric materials occurs because when stress is applied to the surface of the material, the centers of positive and negative charges in the material lattice do not overlap and there is the presence of an electric dipole, and when there is an external stimulus, the disordered electric dipole reverses to form a regular and sequential combination of arrangements, and the centers of positive and negative charges move to the opposite surface of the material, forming a built-in electric field on the two surfaces of the material. This is the phenomenon of polarization [39]. This phenomenon of polarization due to material deformation is called the positive piezoelectric effect [40]. If the direction of the applied force is changed, the polarity of the charge will change. However, for the inverse piezoelectric effect, when the piezoelectric material is pressurized, the electric field force causes the dipole to drive the crystal to deform and produce a polarization phenomenon, to show the stretching behavior of the material surface on a macro-level, which belongs to the inverse piezoelectric effect [41].

### 2.2. Working Principle of PENG

PENG mainly consists of piezoelectric materials and electrodes, and the working principle of PENG is that when piezoelectric materials are subjected to external stress, the positive and negative charge centers of the piezoelectric materials move and a potential difference is formed on the surface of the materials, leading to the generation of transient currents [16,42,43]. To obtain a higher piezoelectric response, it is necessary to go through a polarization method. Due to the positive and negative polarization properties of the material itself, for example, under the same mechanical stress, GaN will produce positive and negative polarization effects with different polarities, but for ceramic or polymer piezoelectric materials, contact polarization and corona polarization methods are commonly used [44]. Due to the irregular “mosaic” of grains in these two groups of materials, the arrangement of electric domains is haphazard and has no polarity, and the electric domains must be oriented in the direction of the electric field by a high-voltage electric field. Contact polarization is to apply high voltage through electrodes on both sides of a piezoelectric material [45]. Corona polarization is based on the principle of corona discharge. At room temperature, the high-voltage electric field will ionize the plasma in the air during the discharge process, and the oxygen atoms with negative charges will be deposited on the surface of the piezoelectric film under the acceleration of the high-voltage electric field. A large number of negative charge ions are deposited on the film to form a potential difference, which makes the electric dipoles reverse, and they are arranged in a certain order. However, for semiconductors, which do not require an electric field to produce polarization, some semiconductors are ferroelectric, and the ferroelectric phase exhibits a spontaneous ferroelectric polarization rate, changing from a paraelectric phase to a ferroelectric phase during the transition from high to low temperature. After the polarization step, the piezoelectric response is increased [46,47].

Since PENG was first proposed by Wang et al. in 2006, many different structures and materials of PENG have been utilized for the preparation of PENG-based energy-harvesting devices [48]. Nowadays, there is a shift from traditional rigid materials such as piezoelectric ceramics (quartz, PZT, and BTO, etc.) to polymeric (PVDF, PDMS, etc.) materials, including composite materials. The flexible design can satisfy more customer requirements and enables the designers to construct customized module functions. To enhance the performance of PENG [49,50,51], researchers have also proposed various structures, such as the vertical growth structure [52,53,54], horizontal growth structure [55,56,57], and stretchable structure [58,59], where the structural and material progress have led to a significant improvement in the performance and extended the application of PENG in human health monitoring, wearable devices, energy harvesting, etc.

### 2.3. Triboelectric Effect

The triboelectric effect can effectively convert mechanical energy into electrical energy based on coupled triboelectrification and electrostatic induction effects. When a material is in frictional contact with another material, the charge between the two materials is transferred, and the charge transfer is accomplished by the thermodynamic movement of electrons from the occupied high-energy state (valence band) to the unoccupied low-energy state (conduction band). To put it simply, a material with a strong ability to obtain electrons will be attracted from another material, and thus electrons gather on the surface of the material, and then when the two materials are separated, an electric potential difference is formed between the two electrodes because a large number of opposite charges have accumulated on the surface of these two materials [60,61,62,63]. The potential difference determines the energy distribution and density of states on the material surface, which is generated by the Fermi energy level of the conducting surface and the effective Fermi energy level of the insulator [64]. The triboelectric layer can generate a large amount of electric charge through periodic contact and separation, and the generated charge can be energy collected after an external circuit. The triboelectric effect is widespread, and many materials in the process of contact-separation produce the corresponding potential difference, so the choice of TENG triboelectric layer materials is very rich, such as wool, melamine, PVC, etc. The polarity of different materials differs, reflecting the different ability of the materials to gain and lose electrons, and the preparation of high-output TENG should use the polarity of the difference between the materials, and thus the friction effect will be stronger [65,66,67,68].

### 2.4. Working Principle of TENG

**Figure 2:**
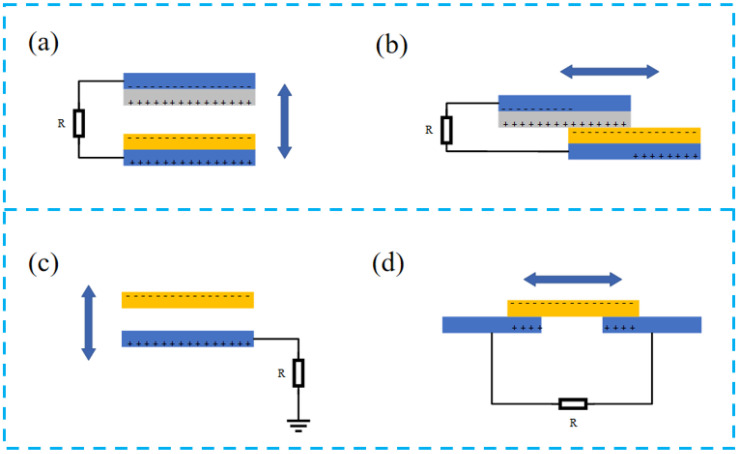
Four fundamental modes of TENG: (**a**) vertical contact-separation mode, (**b**) lateral sliding mode, (**c**) single-electrode mode, and (**d**) freestanding triboelectric-layer mode.

Since TENG was first reported in 2012, much attention has been paid to the collection of randomly generated, irregular, and useless mechanical energy based on Maxwell’s displacement current theory [69,70]. The relative frictional motion of two triboelectric materials generates charges that can be stored through an external circuit for storage. Nowadays, there are four modes of operation of TENG: contact-separation mode, lateral sliding mode, single-electrode mode, and friction-independent mode (Figure 2).

Contact separation is the more common mode (Figure 2a) of TENG. Under the action of external force, the two dielectric materials frictionally separate and contact in the vertical direction, and the charge generated by friction is gathered in the top electrode and back electrode. Periodic contact-separation movement generates a large number of charges and forms an alternation current flow through the external circuit, and thus the mechanical energy is transformed into electrical energy [62].

The sliding mode is structurally the same as the contact-separation mode (Figure 2b), but the direction of motion of the dielectric layer changes from vertical to horizontal, and the charge is generated during relative sliding. The potential difference between the two electrodes changes with the different contact areas of the dielectric layer, and the potential difference forms a current flow, and a regular AC voltage can be generated through the periodic sliding separation [71].

The single-electrode mode is similar to the vertical contact-separation mode (Figure 2c). In this mode, the ground acts as a reference electrode, and the form of charge generation is also generated by contact separation. In this design, the moving part of the TENG is not connected with any electrode, which substantially simplifies the device fabrication process and improves the operation convenience.

Friction-independent mode (Figure 2d) is generated by the motion of the dielectric layer rubbing the symmetrical electrodes and rubbing the contact parts with each other to produce an asymmetrical potential, allowing the current to flow between the electrodes, but the direction of the current depends on the direction of the motion of the dielectric layer, and the friction-independent mode can adopt either sliding-independent TENG or contact-independent TENG [72].

Nowadays, TENG can be used in various aspects, such as environmental monitoring, biomedical and high-voltage applications, etc. [73,74,75,76,77,78]. Due to the many advantages of TENG, such as diverse material choices, easy-to-design specific structures, low cost, high yield, good stability, and excellent output characteristics, it can bring great power to the development of our society.

## 3. Strategy for Enhancing the Performance

From a discrete TENG/PENG nanogenerator to PTCNG, structural design, material selection, and surface modification play an important role in the multi-function, high-output performance, and good stability. The structure design not only increases the consistency of the device structure design, but also greatly reduces the complexity of the device design, making the device more convenient for integrated design. Secondly, for successful coupling of triboelectric and piezoelectric effects, these two properties must be simultaneously considered in the material selection and more attention must be paid on how to evaluate and rank other properties of materials. In many cases, the procedures used in selecting and surface-modifying PENG and TENG materials are still applicable when designing a PTCNG [79,80].

### 3.1. Structural Design of PTCNGs

#### 3.1.1. Electrode Design Strategy

This section describes the fundamental principles of electrode design for integration and how these principles can be used to advance the efficiency of PTCNG. In general, we can always develop PTCNGs with a simplified product design by reducing the number of parts.

**Figure 3:**
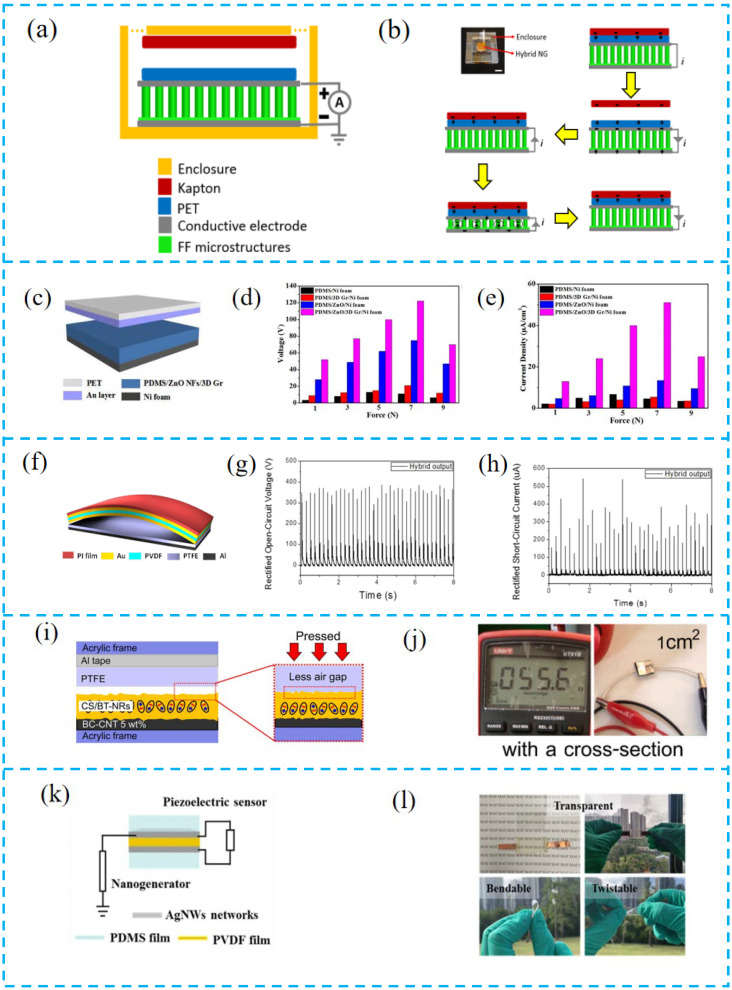
(**a**) Schematic of the structure of the PTCNG. (**b**) Photograph and energy conversion process of the real PTCNG. (**c**) Schematic structure diagram of a PDMS/ZnO nanoflakes/3D graphene-based PTCNG. (**d**,**e**) Comparison of the open-circuit voltage and short-circuit current density under different device structures. (**f**) Schematic structure diagram of an arc-shaped PVDF-PTFE-based PTCNG. (**g**,**h**) Open-circuit voltage and short-circuit of the arc-shaped PVDF-PTFE-based hybrid nanogenerator. (**i**) Schematic diagram of device structure based on the soft BC-CNT conformable electrode. (**j**) Conductivity of a soft BC-CNT electrode. (**k**) Schematic diagram of device structure of PTCNG. (**k**) Transparency, bending, and flexibility of constructed PTCNG. (**l**) Transparency, bending, and flexibility of constructed hybrid nanogenerator.

Nguyen et al. prepared a PTCNG with a single-electrode structure (Figure 3a,b). By sharing the conductive electrodes of PENG, this design does not need to make additional conductive electrodes for single-electrode TENG, thus simplifying the configuration of PTCNG. This strategy of sharing the conducting electrode is universally applicable in the design of PTCNG [81].

Qian et al. prepared a double-electrode PTCNG (Figure 3c–e). The bi-functional layer (PDMS/ZnO NFs/3DGr) has both tribological and piezoelectric characteristics. When the upper electrode is close to the functional layer, the electrostatic induction increases until it contacts the functional layer. As the external force continues to be applied, a current loop will be formed between the upper and lower electrodes. When the pressure is released, all charges will return to their initial state and reach electrostatic balance [82].

Jung et al. designed a three-electrode PTCNG (Figure 3f–h). Gold is deposited on both sides of the piezoelectric layer (PVDF). The other electrode is composed of friction layer (PTFE) aluminum. The middle layer of gold is a common electrode as the driving electrode of the two functional layers. Through this three-layer electrode design, the TENG part works in the contact-separation mode, and the device shows an impressive output enhancement, with an open-circuit output voltage of 370 V and a current density of 12 μAcm^−2^, and an output power density of 4.44 mWcm^−2^ [83].

Pongampai et al. introduced bacterial cellulose/carbon nanotube composites to make soft electrodes (Figure 3i,j), which can reduce the gaps that are formed due to the surface microstructure between the electrode and the dielectric material. Such a design can both increase the contact efficiency of the TENG part and improve the charge transfer density between the piezoelectric layer and the electrode. As a result, the contact efficiency (η) reached 91.67% and the output current density increased 1.1 times [84].

Yu et al. made transparent and flexible hybrid nanogenerators (Figure 3k,l) by welding silver nanowires (AgNWs) as electrodes. Due to the recrystallization and merging of AgNWs at the contact position, the conductivity of AgNWs electrodes was greatly improved, which reduced the contact resistance in both the TENG and PENG parts, while the transparency of AgNWs/PVDF reached 71% with high flexibility, electrical output, and sensing performance [85].

The preparation of electrodes is one of the factors for sensors with high-output characteristics. More research is built on the use of traditional metal electrodes, which are not only costly but can also cause some metal pollution, and research on electrodes with ultra-flexible and high conductivity is urgently needed.

#### 3.1.2. Overall Structure Design of PTCNGs

**Figure 4:**
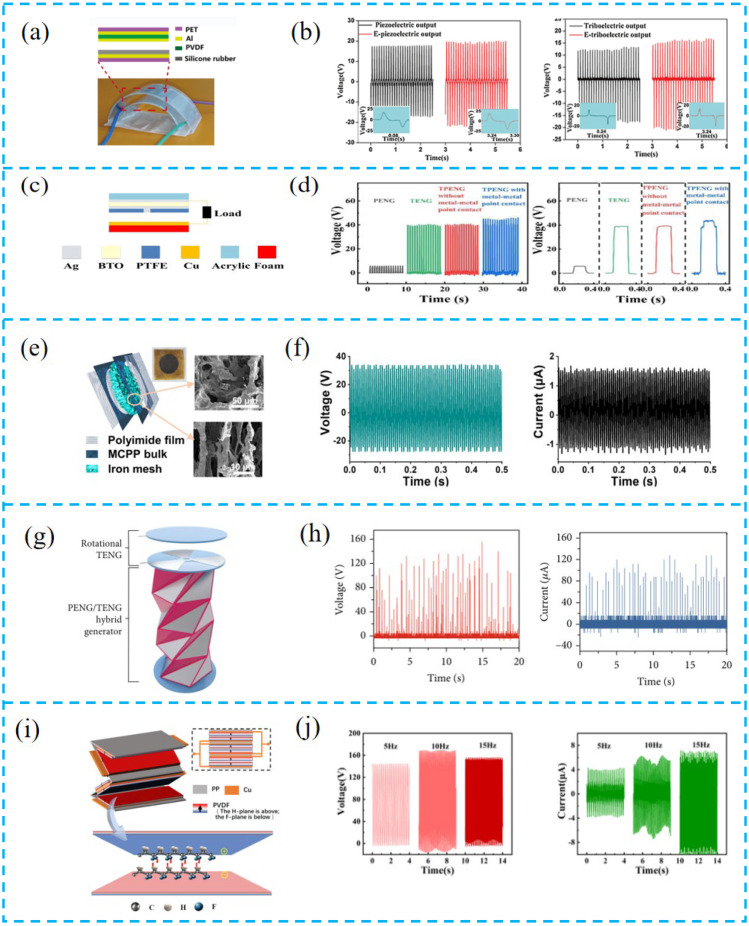
(**a**) The photo image and schematic illustration of the device. (**b**) Open-circuit output voltage and short-circuit output currents of the piezoelectric unit and triboelectric unit, respectively. (**c**) Structure of the PTCNG. (**d**) Open-circuit voltage and short-circuit current density of different device structures. (**e**) Structure diagram of the MCPP ANG and SEM images of the aerogel bulk. (**f**) The optimal output voltage and current of the quenched MCPP ANG. (**g**) Structural diagram of PTCNG. (**h**) Open-circuit voltage and short-circuit current output of the PTCNG. (**i**) 3D schematic diagram of the PTCNG. The inset map is the electrode connection diagram. (**j**) Open-circuit voltage and short-circuit current output of the PTCNG under different frequencies.

A well-designed structural integration strategy is increasingly valuable as the development of PTCNG dramatically increases the reliability and improves the performance of the device. Zhu et al. designed the generator with a D-arch structure (Figure 4a,b). The top PET film not only plays a supporting role, but also improves the output performance of the piezoelectric layer. In addition, the finite element simulation of PVDF film established through COMSOL software confirmed that the PET film with a thickness of 0.3 mm is the most appropriate. When a force of 3 N is applied to the top of the 0.3 mm PET film, the maximum piezoelectric potential is 44.15 V [86].

Zhu et al. drilled a small hole with a diameter of 1.2 mm on the PTFE film (Figure 4c,d). The hole acts as a conductive channel, which makes it easier to form metal–metal point contact between the Ag electrode in the middle of the device and the Cu electrode at the bottom, increasing the charge transfer rate. This innovative structural design also provides a feasible method to distinguish the piezoelectric and triboelectric effect processes [87].

Yu et al. prepared a PTCNG with beam-shaped porous MWCNTs/P(VDF-TrFE) aerogel and a PDMS valve structure (Figure 4e,f). This design promotes the diffraction and scattering of sound waves inside the hole wall, enhances its vibration and friction, and thus produces enhanced output. In addition, the flexible PDMS valve is designed to imitate the human tympanic membrane, which has a certain pressurization effect and good frequency response characteristics. The two structures constitute a built-in composite acoustic resonator. The optimal output voltage and current of the tested device under 150 Hz and 115 dB acoustic stimulation are 34.4 V and 1.74 μA. The corresponding peak-to-peak voltage is 62.0 V, and the power density is 11.62 mW/m^2^ [88].

Inspired by ancient origami, Chung et al. prepared a PTCNG based on triangular cylinder origami, which is composed of vertical TENG, rotary TENG, and PENG (Figure 4g,h). The cylinder is composed of multiple triangles, and the base is a pentagon. The design of this structure can maximize the requirements of PENG’s high mechanical deformation and TENG’s large surface area. The single-layer surface contact area of the cylinder is 1.38 times that of the plane and improved the output [89].

Wang et al. adopted the head-to-head parallel design (Figure 4i,j). By connecting piezoelectric films with the same polarity, triboelectric elements were added to the gap between two contact films. In most studies on piezoelectric fiber films, the high performance is attributed to piezoelectric properties, while their triboelectric contributions are ignored. Such a design realizes the synergistic effect of triboelectricity and piezoelectricity. The output performance of the films fabricated by head-to-head parallel assembly is about four times that of conventional series connection [90].

The overall structural design of the device is more about increasing the high mechanical deformation rate of the PENG and the friction area ratio of the TENG, and it is often easier to ignore how to enhance the charge transfer rate and reduce the charge loss.

### 3.2. Material Selection

**Figure 5:**
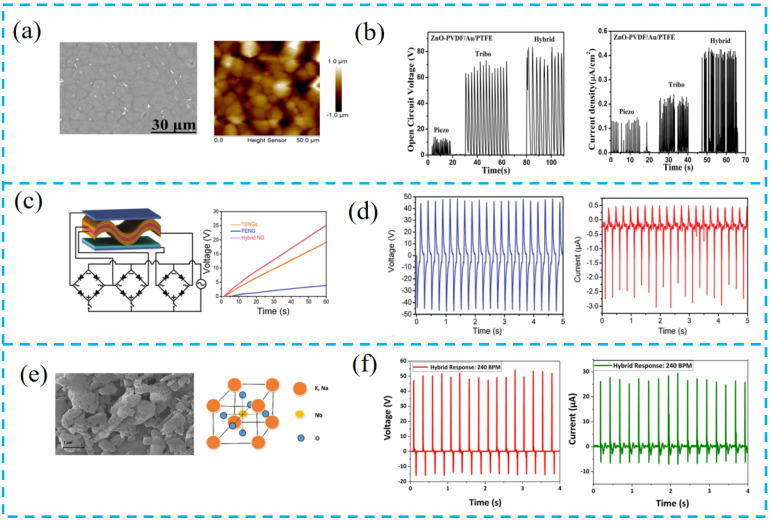

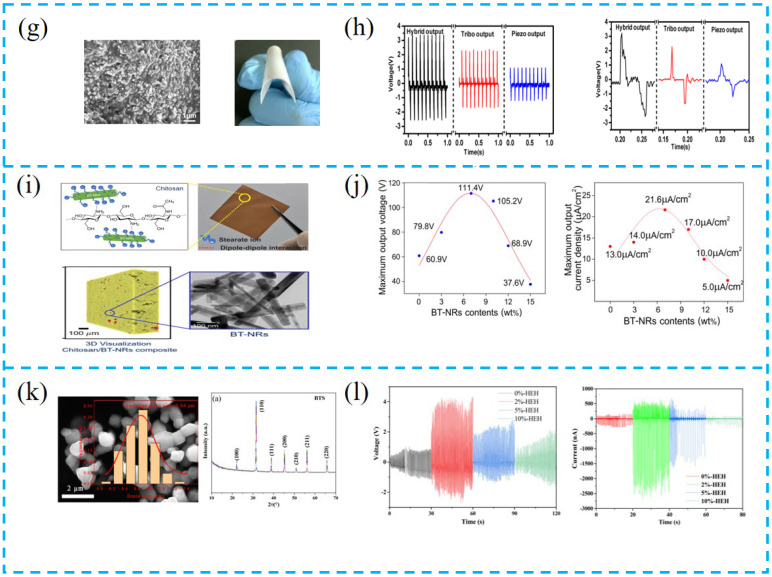

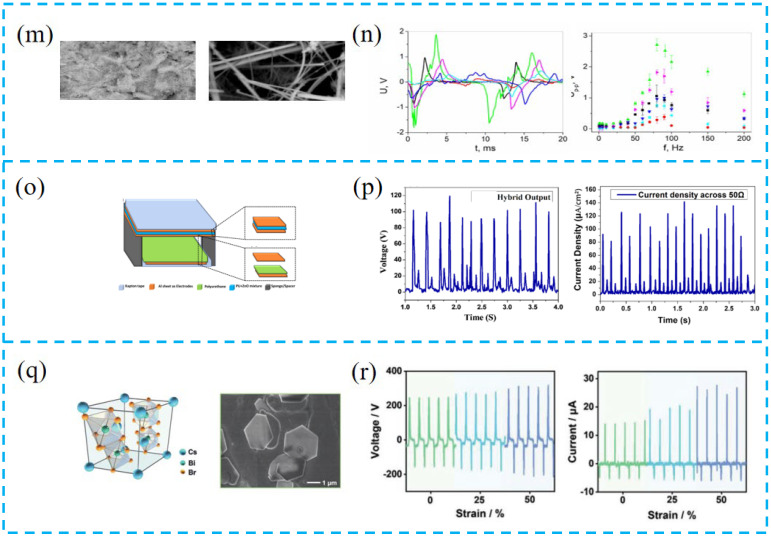
(**a**) SEM and AFM images of ZnO-PVDF film and schematic diagram of PTCNG. (**b**) Rectified open-circuit voltage and short-circuit current density of PTCNG. (**c**) The circuit diagram of the PTCNG for lighting up LEDs and charging ability test of the PTCNG. (**d**) The output voltage, output current, and instantaneous power changes with the increasing load resistance for the PENG. (**e**) Perovskite crystal structure of KNN and SEM image of KNN powder. (**f**) Output voltage response and output current response of the EF-attached PTCNG at a 240 BPM tapping frequency. (**g**) Photograph of the prepared BTO/PDMS composite film and cross-sectional SEM image of the composite film. (**h**) Output voltage under the three testing modes: hybrid, triboelectric, and piezoelectric. (**i**) Different views of 3D virtualization by SRXTM analysis of CS/BT-NRs 7 wt.% composite film, with an inset of the TEM image for BT-NRs. (**j**) Average maximum voltage output and average maximum current output with a tendency for varying amounts of BT-NRs fillers (0, 3, 7, 10, 12, and 15 wt.%) and composited films. (**k**) XRD pattern and the SEM image of the BTS particles (the inset shows its grain size distribution). (**l**) The open-circuit voltage and the short-circuit current of the HEH with different BTS particles’ content. (**m**) The SEM image of the SbSeI nanowire. (**n**) Voltage signals recorded for shaker tip excitation at an 80 Hz frequency and spectra of peak-to-peak voltage generated by driving the shaker with a rectangular signal for an unloaded device. (**o**) The schematic of the proposed PTCNG. (**p**) Rectified output voltage and current density of PTCNG. (**q**) Unit cell crystal structures of Cs3Bi2Br9 and SEM images of pure Cs3Bi2Br9 thin film. (**r**) The output voltage and current of LPPS-NFC-based TPENG under 0%, 25%, and 50% strain.

The selection of functional layer materials is very important for PTCNGs. While structural materials are selected for their load-bearing capacity, functional materials are selected for the nature of their response to mechanical, electrical, magnetic, optical, or chemical stimuli, thus endowing the PTCNGs with multifunction and robustness. For instance, Singh et al. added ZnO into PVDF to prepare PTCNGs (Figure 5a,b). The addition of ZnO induces the increase of β-phase in PVDF and leads to the enhancement of piezoelectricity. Moreover, it makes the surface roughness of ZnO-PVDF nanocomposite film increase, which improves the surface charge density for triboelectrification, and the output of the tested composite film-prepared devices is 2.5 times higher than that of bare PVDF composite nanogenerators [91].

Chen et al. used electrostatic spinning to prepare P(VDF-TrFE) nanofiber films (Figure 5c,d). The P(VDF-TrFE) material has higher piezoelectricity and flexibility than conventional PVDF, which can increase the contact area with electrodes and can collect more charges and enhance the output of the generator. Under the stress of a frequency of 4 Hz, the peak output and current generated by the piezoelectric part are 96 V and 3.8 μA, which is about two times the initial output. Additionally, the performance of the triboelectric part was improved by 8 and 16 V with the assistance of the piezoelectric potential [92].

Inorganic materials have greater piezoelectric properties. Abdullah et al. used potassium sodium niobate (KNN) combined with porous carbon nanotubes (MWCNT) into PVDF matrix (Figure 5e,f). Since KNN has a large piezoelectric response, ferroelectric properties, high Curie temperature (>400 °C), and is suitable for low-cost and lightweight requirements, it was experimentally demonstrated that when the specific gravity of KNN reached up to 5%, the nanogenerator fabricated using this composite film had a much higher output performance [93].

Suo et al. combined BaTiO_3_ with PDMS to form a composite structure (Figure 5g,h). When the BTO content was 20% and the film thickness was 100 μm, the device showed excellent output performance, and the addition of BTO was the main factor affecting the piezoelectric output, and the preparation of composite films using this material led to an increase in the dielectric constant and the charge density of PDMS. The PTCNG prepared using BTO material has the advantages of simplicity, low cost, and high performance [94].

Pongampai et al. extracted the natural polymer chitosan from animal shells and hybridized it with lead-free BaTiO_3_ nanorods (Figure 5i,j). Due to the natural degradability, biocompatibility, non-toxicity, and easiest processing of chitosan into thick and thin film forms, the huge number of primary hydroxyl (–OH) and secondary amine (–NH_2_) groups tends to release electrons, thus generating positive zeta potentials, and offering proper matric cation trapping for modification, enhancing the output of TENGs [84].

Shen et al. used a mixture of Ba(Ti,Sn)O_3_ and P(VDF-CTFE) materials to prepare the piezoelectric layer (Figure 5k,l). The addition of Sn^4+^ further increased the BaTiO_3_ piezoelectricity, and the peak open-circuit voltage was 4.16 V when the BTS particle content was 2 wt.%. A further increase of the BTS content would lead to a decrease in the output voltage, and the performance output decreased due to the high conductivity of BTS particles, and the insulating properties of the composite are weak because the BTS particles are not uniformly dispersed [95].

Toron et al. fabricated a compressed antimony iodide (SbSeI) PTCNG (Figure 5m,n). Its high surface triboelectricity and the highly reactive surface area allow for the induction of electrostatic charges, and the prepared device produced significantly higher voltage per surface area and force unit (15.8 V/(N×cm^2^)) compared to similar reports of other PTCNGs [96].

Ali et al. used UV-curable polyurethane (PU) and ZnO composites to prepare PTCNGs (Figure 5o,p), combining PU with ZnO powder to make full use of the advantages of biocompatibility, high durability, and toughness for the fabrication of PENG and TENG. In the synthetic planar band, self-healing PTCNG showed an open-circuit voltage of 120 V and a current density of 140 μA/cm^2^, which means that it can be widely used in portable devices, health monitoring, and energy harvesting in the future [97].

Jiang et al. prepared a stretchable, breathable, and stable nanofiber composite (LPPS-NFC) by electrospinning lead-free chalcogenide/(PVDF-HFP) and SEBS (Figure 5q,r), where Cs_3_Bi_2_Br_9_ is an effective electron acceptor for polymer chain crystallization and the good energy level matching between Cs_3_Bi_2_Br_9_ and PVDF-HFP improves the electron transfer efficiency and reduces the charge loss, thus facilitating the electron capture process. The devices can operate continuously for up to five months, setting a record output voltage for halide chalcogenide-based nanogenerators, extending the applications of halide materials and opening new avenues for future research on electronic skin and wearable devices [98].

The choice of materials is generally ceramics with high-voltage electrical coefficients for doping, and piezoelectric ceramics are not environmentally friendly and can easily cause harm to human health if made into flexible wearable devices. There is a trend to find natural extracts with biocompatible properties to prepare sensors.

### 3.3. Surface Modification

Surfaces are the boundaries with the external environment and the interface, where the electrode material works and behaves to present good functioning and the object made thereof. Surface modification that tailors the surface property is therefore needed to transform them into highly efficient energy-harvesting and sensing films and has become an important part of the nanogenerator research.

**Figure 6:**
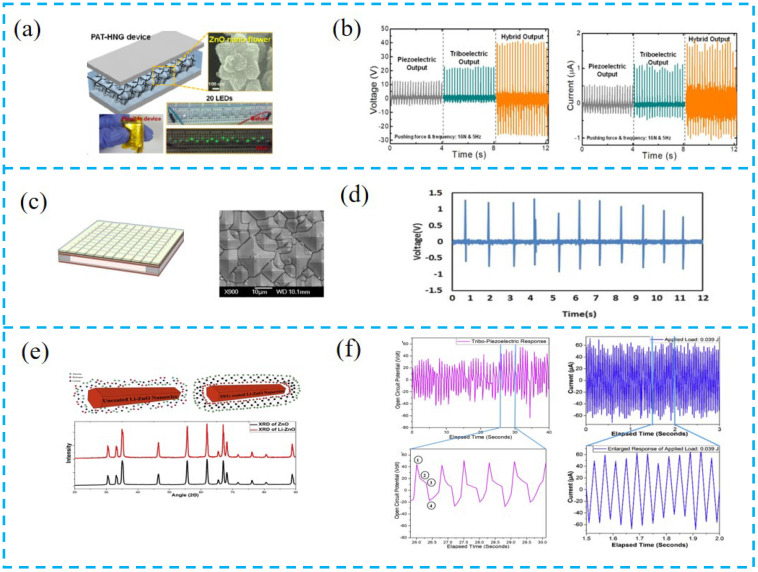

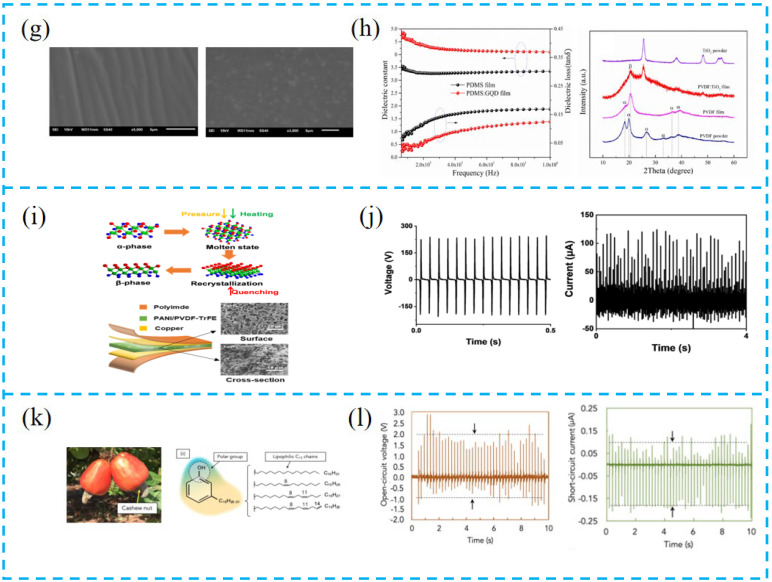
(**a**) Schematic diagram for the PAT-HNG device. (**b**) Output voltage and current of the device under piezoelectric, triboelectric, and hybrid modes. (**c**) Schematic diagram of the device and SEM image of NEFS fiber arrays deposited on the PCB substrate. (**d**) Output voltage of PTCNG. (**e**) XRD diagram of ZnO and Li-ZnO. (**f**) Response of PTCNG and current response of PTCNG under applied load of 0.039 J and enlarged response of applied load of 0.039 J. (**g**) Surface SEM images of PVDF films. (**h**) Dielectric constant and dielectric loss of PDMS and PDMS: GQD films and XRD pattern of PVDF films before and after modification with TiO2 nanoparticle. (**i**) The phase transformation process of PVDF-TrFE under hot pressing and liquid nitrogen quenching, structure diagram of the PTNG, and surface and cross-section SEM images of the aerogel bulk. (**j**) The optimal output voltage and current of the quenched PANI/PVDF-TrFE PTNG. (**k**) Cashew nut as a source for the production of cardanol oil, and chemical structure of cardanol with the indication of the polar group and lipophilic chains. (**l**) Open-circuit voltage and short-circuit current generated by the PTCNG, under finger tapping (~2 N, ~5 Hz) and with a 10 MΩ probe.

Kim et al. prepared a flower-like ZnO nanostructure by chemical precipitation and combined ZnO, MWCNT, and PDMS (Figure 6a,b). The high roughness introduced by the ZnO nanoflowers on the PDMS surface can be used to enhance the triboelectric potential while increasing the piezoelectric potential and the degree of polarization [99].

Fang et al. used a surface texture transfer technique to transfer texture patterns onto thin films to prepare micro-nanostructures (Figure 6c,d). This structure can increase the effective contact area, capture more charges, and improve the output of triboelectricity. Devices with an area of 30 × 25 mm were prepared with an open-circuit voltage of up to 1.2 V and a short-circuit current of up to 30 nA, which can also be used as an acoustic detection device to capture small vibrations [100].

Doping technology is an important direction in the research of thin-film devices, where two or more materials are doped to improve the output performance of the device by changing the properties of the materials through chemical interactions. Chowdhury et al. inserted ZnO nanowires with surface modification into the PVDF matrix (Figure 6e,f). The effect of ZnO is to induce an increase in the β-phase in PVDF, which enhances the bipolar ordering of PVDF, due to the increase in the negative charge on the NW surface by local electrostatic interactions of the dipoles of PVDF, and this interaction of chemical groups can reduce the necessity of polarization. The devices prepared by surface modification have a stronger piezoelectric response and lower dielectric impedance [101].

Yang et al. used a double-doping technique to dope the piezoelectric and friction layers simultaneously (Figure 6g,h), using graphene quantum dots (GQD) and titanium dioxide (TiO_2_) nanoparticles to further modify PDMS and PVDF. The addition of GQD improves the dielectric properties of the film and increases the effective contact area with the electrode, and the addition of TiO_2_ induces an increase in the β-phase in PVDF, which increases the piezoelectric properties and leads to a greater improvement in device performance [102].

Yu et al. used a new strategy of liquid nitrogen mutagenesis and doping technology to prepare high-performance PTCNGs (Figure 6i,j). The liquid nitrogen quenching method allows the β-phase to be increased to 71%, which is essentially due to the rapid cooling of liquid nitrogen from the bottom to the top, resulting in the conversion of disordered molecules in molten α-PVDF into ordered β-phase, in addition to causing the rapid cooling and recrystallization of PVDF, resulting in a highly uniformly oriented β-phase. Polyaniline (PANI) doping enhances the mesoporosity of composite films and traps more charge [103].

As the research on surface modification deepened, many researchers searched for low-cost, all-natural modifiers. Mariello et al. proposed an oily component cashew nut shell liquid (CNSL) in cashew nut agricultural products (Figure 6k,l). Cashew nut phenolic oil as a plasticizer can form a dense fiber network and improve the flexibility and dielectric constant of electrospun PVDF, which facilitates contact charging during friction due to its lipophilic alkyl chains [104].

Surface modification is one of the means to improve device performance, and doping techniques to alter the film morphology to increase friction are commonly used. Today, there exist many researchers exploring low-cost, purely natural modifiers to modify the piezoelectric and frictional properties of thin films to further improve device performance output.

## 4. Application of PTCNGs

With tunable flexibility, adaptivity, and enhanced output and sensing performance, PTCNGs have been widely used in energy conversion and physiological monitoring systems.

### 4.1. Tactile Sensors Based on PTCNG

PTCNG are not only capable of self-powering the system to make it continuously operational but can also be used as sensors to detect various tiny signals in human motion and disease monitoring. So far, tactile sensors can fuse different sensing mechanisms (piezo-resistive, capacitive, piezoelectric, and frictional electric) and materials to make various wearable tactile sensors, while smart sensor devices with practical functions such as biocompatibility, degradability, self-healing, and self-energy supply come into being. In addition, tactile sensors are effectively integrated with related functional components to create wearable systems with good flexibility, spatial adaptability, and functionality [105,106].

#### 4.1.1. Tactile Sensor for Motion Monitoring

**Figure 7:**
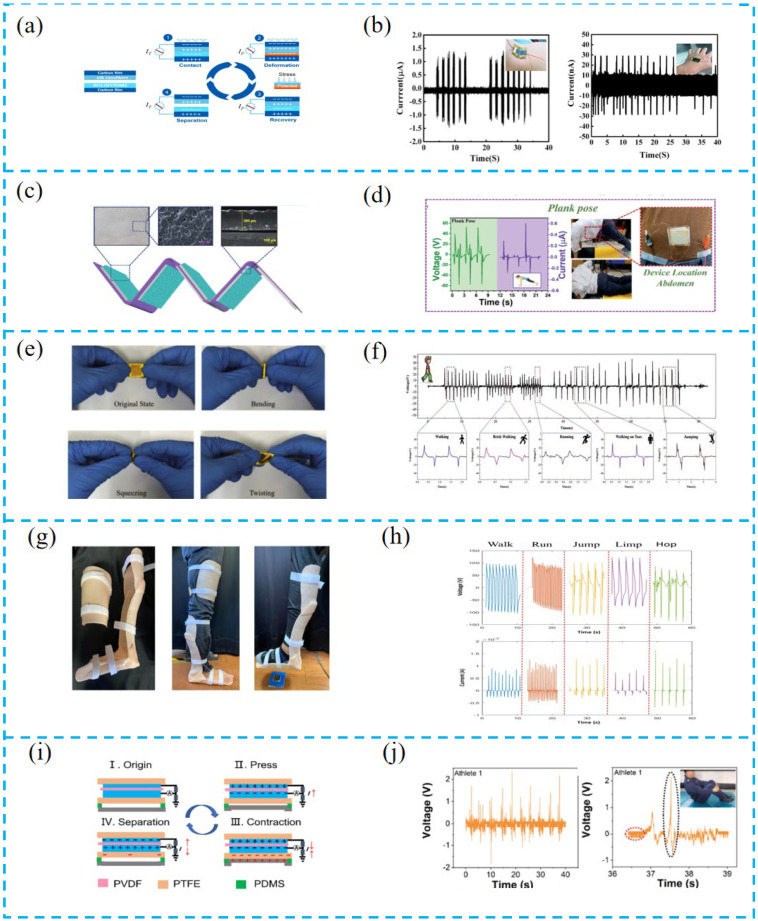
(**a**) Device configuration of PTCNG and working mechanism of PTCNG. (**b**) Output current of the PTCNG for real-time posture monitoring and harvesting body-motion energy. Inset shows the corresponding photography images for the tests. (**c**) Schematic representation of multi-stack PTCNG along with the digital picture of rough film, surface morphology of rough film, and cross-sectional image of the film. (**d**) Demonstration of body activity counter with various yoga movements for calculating the calories burnt. (**e**) Photographs of the HPTS under different deformations. (**f**) The HPTS could monitor the human posture. (**g**) Response to different types of action modes while wearing a leg rehabilitation device. (**h**) The data on the output of five different action modes (walk, run, jump, limp, hop) while wearing a rehabilitation device. (**i**) Coupling mechanism of TENG and PENG. (**j**) Output voltage and details of athlete 1’s 301C diving motion, and output voltage and details of athlete 2’s 301C diving motion. Wireless Bluetooth transmission system.

Liu et al. mounted a PTCNG on the human arm (Figure 7a,b), which can generate an output current under both bending and stretching with a maximum output current of 1.5 μA. Due to the narrow gap of the triboelectric pair and the high sensitivity of the ferroelectric composite film, the device can be placed on the back of the hand to detect the weak body motion energy during clenching and stretching of the fist, enabling the collection of energy from daily movement energy [107].

Sahu et al. prepared a PTCNG using the high-voltage electrical properties of lead-free ceramic (BCZT-BH) (Figure 7c,d). This device is integrated with an IR transmitter, receiver, and self-powered wireless IR communication module for human yoga counting, and the output of the device can also reach 60, 120, and 45 V in three postures. This device effectively counts the repetitive movements of the exerciser and the effective degree of the body, providing a possibility for commercializing self-powered counters in the future [108].

Yu et al. proposed a piezoelectric-triboelectric hybrid sensor (HPTS) based on PZT and PDMS. This tactile sensor can be installed at human joints to monitor human motion posture, record multiple motion states, detailed motion physiological information, and obtain stride frequencies of various gait, which shows the excellent ability of HPTS to monitor human motion [109].

Lu et al. proposed a self-powered multifunctional sensor on the coupled piezoelectric/triboelectric effects (SPMS) to test these movements (walking, running, jumping, limping, hopping) of people (Figure 7g,h). To maximize the use of the new sensor as a gait sensor for monitoring human movement characteristics, it was combined with machine learning to identify human biomechanical characteristics, recording useful waveforms from the SPMS and obtaining pattern recognition. The results showed that individual sequential gait SPMS signal recognition accuracy reached 81.8% [110].

Zhu et al. reported a piezoelectric-triboelectric electrokinetic sensor (PTSS) consisting of TENG, PENG, and flexible transparent stretchable self-healing hydrogel electrodes (Figure 7i,g). The PTSS has a wide monitoring range due to the coupling of piezoelectric and triboelectric effects. It can be used to monitor multidimensional movements of the human body such as bending, twisting, and rotation, including the spiral pulling action of table tennis and the 301C technique of diving. This PTCNG can be used as a wearable motion monitoring sensor, providing a new strategy in the field of sports, motion monitoring, and human–computer interaction [111].

#### 4.1.2. Tactile Sensor for Disease Monitoring

**Figure 8:**
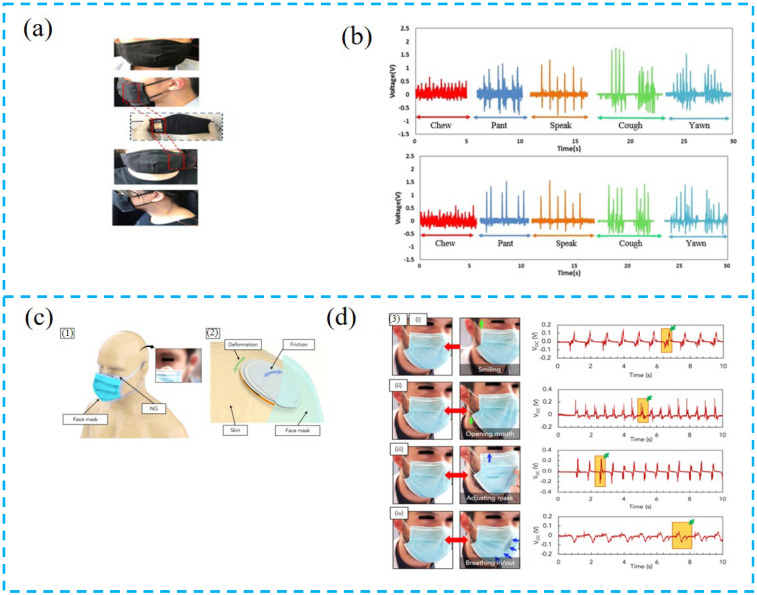

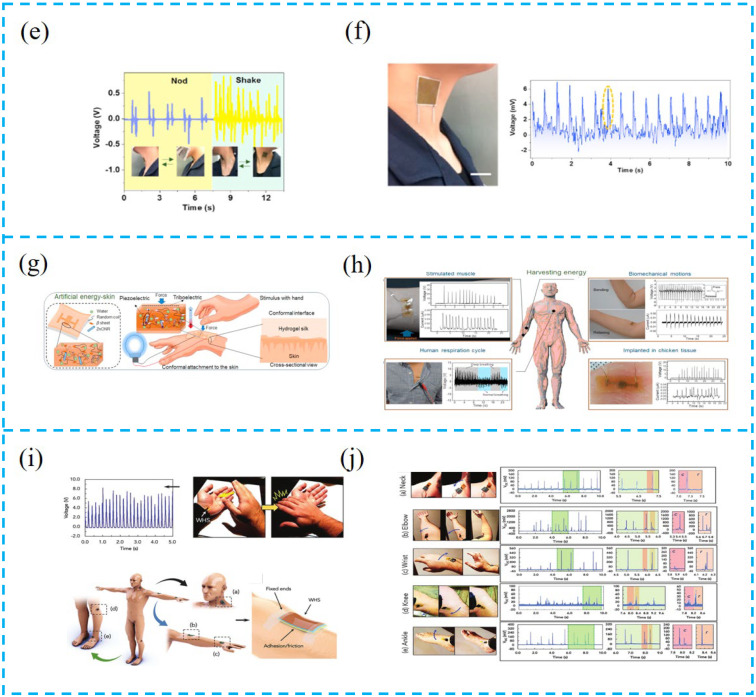
(**a**) Response to different types of mask vibrations for two different testers. (**b**) PTHS output voltage, with various facial movements of the test subject. (**c**) (**1**) Three-dimensional (3D) illustration and real photo of the applicability of the hybrid PENG underneath the surgical face mask. (**2**) Concept of the motional energy sources harvested by the hybrid PENG. (**d**) (**3**) Open-circuit voltage signals generated by the hybrid PENG for specific movements reproduced at ∼1 to 1.5 Hz: (**i**) smiling, (**ii**) mouth-opening, (**iii**) adjusting the mask, and (**iv**) normal breathing in/out. (**e**) The voltage signals of head movements. (**f**) Photograph demonstrating the E-skin as a wearable self-powered pulse monitoring sensor. (**g**) Schematic illustration to show the concept and working principle of artificial EG-skin using silk hydrogel. (**h**) Forearm by tapping the hand to stimulate the muscle, elbow by bending and releasing, chest for monitoring respiration cycles, and embedded in chicken breast tissue. (**i**) Open-circuit voltage of the HS under hand tapping frequency of ~5 Hz and force of ~5 N on the WHS applied on the human wrist (as in the photos), and 3D representation of the human body and the positions of the WHS for sensing human gestures. (**j**) Open-circuit voltage of the WHS for monitoring movements of (**a**) neck, (**b**) elbow, (**c**) wrist, (**d**) knee, and (**e**) ankle.

Tactile sensors can be applied to disease monitoring. Fang et al. constructed a human–computer interaction command control system based on PTCNGs (Figure 8a,b), and the combination of advanced technology (LSTM), which was integrated into a smart mask, recognized personal human characteristics through facial muscle dynamics, and the recognition rate of chewing, gasping, talking, coughing, and yawning can reach 87.9%. This device can provide a monitoring method for respiratory diseases in the future [100].

Mariello et al. prepared a biocompatible hybrid device (Figure 8c,d). Due to the strong electrostatic property of the membrane and the plasticization of cardanol, the PTCNG has ultra-sensitive electromechanical conductivity. This device reveals the potential applications of intelligent masks and coupling energy-collection devices in the future. It can not only be used for anti-infection protection, but also to provide sensors or active antibacterial/viral devices [104].

Zhu et al. reported the electronic skin with excellent perception ability (Figure 8e,f), which can measure and distinguish the sense of contact and non-contact, such as sense of click, distance discrimination, breath detection, head motion perception, vocal cord vibration recognition, and some physiological signal monitoring, further expanding the application scope of electronic skin detection and providing better diagnostic information for predicting Parkinson’s disease and cardiovascular disease [112].

Since the electronic skin is in direct contact with the human body, it is a trend to use green and non-polluting materials to avoid the toxicity of the material causing harm to the human body. Gogurla et al. reported the electronic skin based on the engineering silk protein hydrogel (Figure 8g,h). The triboelectric and piezoelectric effects are synergistic, which are used to monitor human muscle strain, respiratory activity, and can be used as an implantable electronic device, generating high power (~1 mW/cm^2^), enough to turn on the blood oxygen meter to monitor human health [113].

Mariello et al. prepared a highly sensitive and ultra-flexible PTCNG based on AlN and polystyrene encapsulation (Figure 8i,j). The device is in direct contact with the skin through an ultra-flexible patch covered with an ultra-thin friction parylene film on both sides, and the device exhibits a sensitivity of 59.4, 160, and 3.7 mv kPa^−1^ at pressures of 0~50, 50–120, and 120–400 kPa, respectively. In addition, the sensor can be applied to the monitoring of human heart rate, blood pressure wave, voice vibration, or body movement to detect micro-friction phenomenon and ensure the stability and repeatable recognition of biological signals [114].

The main challenge of PTCNGs today is that they can only measure dynamic stresses, and strategies for preparing multimode sensors are relatively easy. However, high crosstalk between multiple sensing modes hinders them from detecting both static and dynamic stresses, and they must have self-powered, fast response, and high-sensitivity capabilities. As mentioned above, it is important to accelerate the research of PTCNGs [115].

### 4.2. Energy-Harvesting System Based on PTCNG

#### 4.2.1. Clean Energy Harvesting

**Figure 9:**
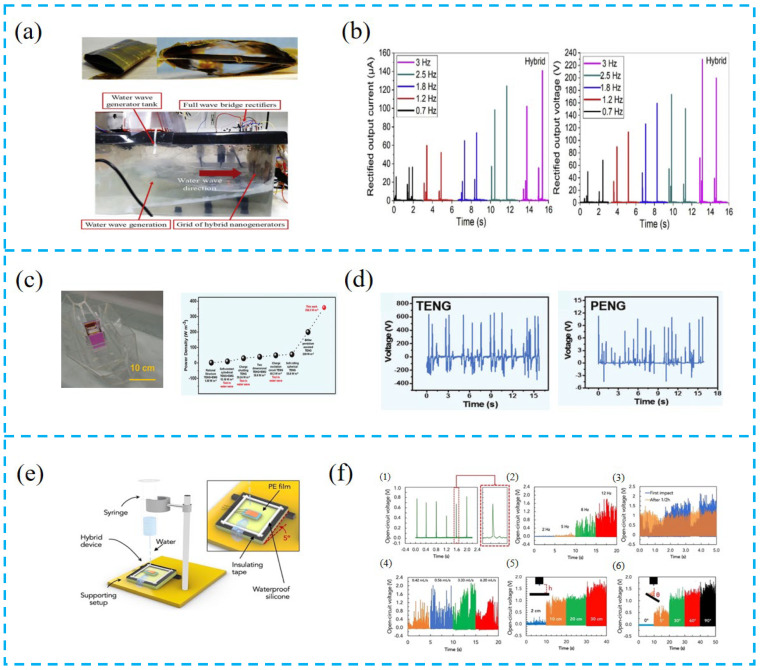

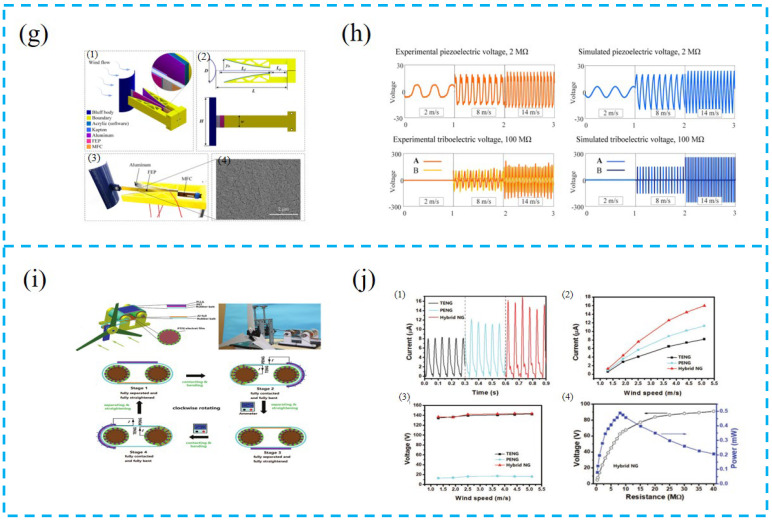
(**a**) Digital photo and cross-sectional digital photo of the hybrid nanogenerator. (**b**) Average instantaneous rectifier output current and output voltage at different frequencies. (**c**) Photo of a boat with incorporated BCHNG modules in a water tank, and state-of-the-art performance of the device structure with the wave energy harvesting. (**d**) Output performance of different parts of the hybrid nanogenerator plotted in simulated water waves. (**e**) 3D representation of the custom-made setup for simulating rainfalls. (**f**) (**1**) Open-circuit voltage of the HNG under the impact of single 100 µL water droplets. (**2**) Open-circuit voltage of the HNG under the impact of single raindrops at different frequencies in the range of 2–12 Hz. (**3**) Open-circuit voltage of the HNG under simulated rainfalls after the first impact with water and after 1/2 h of continuous water falling. (**4**–**6**) Open-circuit voltage of the HNG under simulated rainfalls for (**4**) different flow rates, (**5**) different heights, and (**6**) different inclination angles of the HNG with respect to the vertical direction. (**g**) (**1**) Design of a synergetic PTCNG wind energy harvester (SHPTWEH), (**2**) its geometric configuration, (**3**) the prototype, and (**4**) an SEM image of the FEP used in the device. (**h**) The voltage response of the SHPTWEH at different wind speeds obtained from experiments and simulations, respectively. (**i**) Schematic diagram of the structure, photograph of the hybrid nanogenerator (**left** and **right**), and the working principle of the PTCNG. (**j**) (**1**) Real-time I_sc_ measurement of the E-TENG, PENG, and hybrid NG at a wind speed of 5.1 m s^−1^. (**2**) Rectified I_sc_ as a function of wind speed for the E-TENG, PENG, and hybrid NG. (**3**) Voc of the E-TENG, PENG, and hybrid NG as a function of wind speed. (**4**) The output voltage and output power as a function of load resistance for the hybrid NG.

It is a trend to use PTCNGs to collect clean energy (low-frequency mechanical energy, wind energy, water wave energy, etc.) in the future. Jurado et al. designed a coastal wave energy collection system (Figure 9a,b). When the simulated actual wave frequency is 0.7–3 Hz, the output performance is improved by about 2 times compared to the use of a single nanogenerator. In addition, this equipment can be used in the power supply network of the artificial intelligence sensor system. By establishing a large PTCNG interface, it can produce about 21.61 W of high-output power, and the energy conversion efficiency can reach 30.22% [116].

Zhang et al. prepared a dual-pendulum-coupled PTCNG (BCHNG) module (Figure 9c,d). This module collects the kinetic energy and gravitational potential energy of water waves. The peak power density of the module reaches 358.5 Wm^−3^, which is much higher than that of the landmark dual-pendulum-assisted TENG (200 Wm^−3^). Although the load impedances of the two generators are not the same, compared with a single generator, the simple superposition of PTCNGs can effectively improve the collection efficiency of water wave energy, and establish a foundation for self-powered ocean-sensing detection using clean energy in the future [117].

Mariello et al. used the hybrid device to collect raindrop energy and water wave energy (Figure 9e,f). A single raindrop (100 µL) can produce a maximum voltage of ~0.8 V. When the rainfall is 3.33 mL/s, the output voltage is as high as ~2.2 V. Since a single nanogenerator has many disadvantages, such as low-output voltage and current, different internal impedance, and is not suitable for all applications, the preparation of this hybrid device increases the ability to collect energy from different water transfer sources (such as impact/breakwater, raindrops, buoyancy waves) and expands the application range of the sensor [118].

Although the traditional wind power generation equipment can achieve good energy conversion efficiency, it has the disadvantages of huge volume and high maintenance costs. However, the application of PTCNGs can not only collect wind energy, but also power small electronic devices. Wang et al. designed the boundaries of the triboelectric nanogenerator to improve the performance of the device. These boundaries not only limit the maximum deformation of the beam under high wind speeds and avoid damage to the device, but also increase the vibration frequency of the device (Figure 9g,h). The experiment shows that this device can work stably at low and high wind speeds, expand the working wind speed range, improve the conversion efficiency of wind energy to electric energy, and can be used in small wind power generation systems in the future [119].

Zhang et al. used the PTCNG to form a wind power generation system (Figure 9i,j). At the wind speed of 5.1 m s^−1^, the open-circuit voltage and short-circuit current of the PTCNG are 140 V and 16 µA, respectively, the maximum output power is ~0.49 mW, and the matching resistance is 8 MΩ, which is 22% higher than the output power of E-TENG. The PTCNG can charge the lithium battery to 2.9 V within 8 h, which is used to drive the infrared remote LED lamp and control the switch of the lamp. It effectively uses the wind energy and increases the recycling of clean energy [120].

PTCNGs can only collect low-frequency mechanical energy, wind energy, water wave energy, etc. If its application is made wider and the practicality is accelerated, it is important to explore the stability and repeatability in high-frequency energy collection, not only to strengthen the study of material selection and preparation processes, but also to meet the reasonable design of the whole energy collection system.

#### 4.2.2. Self-Powered System Based on PTCNG

**Figure 10:**
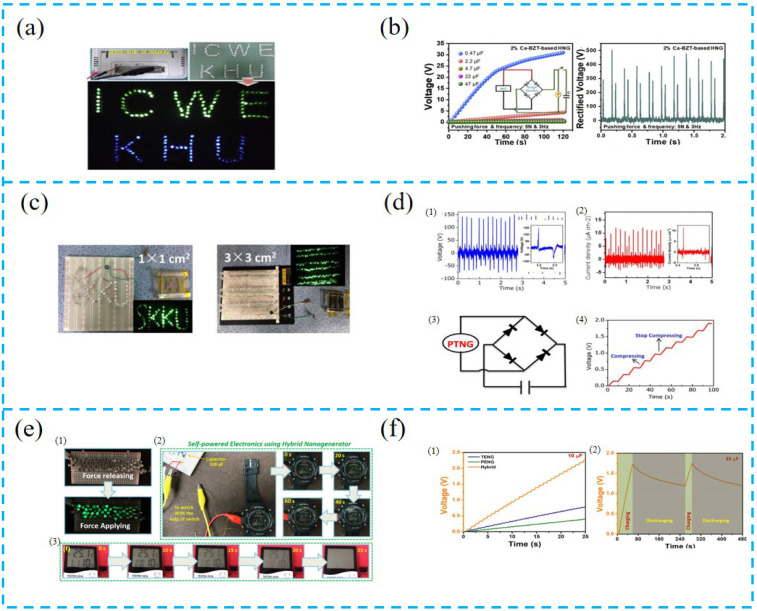

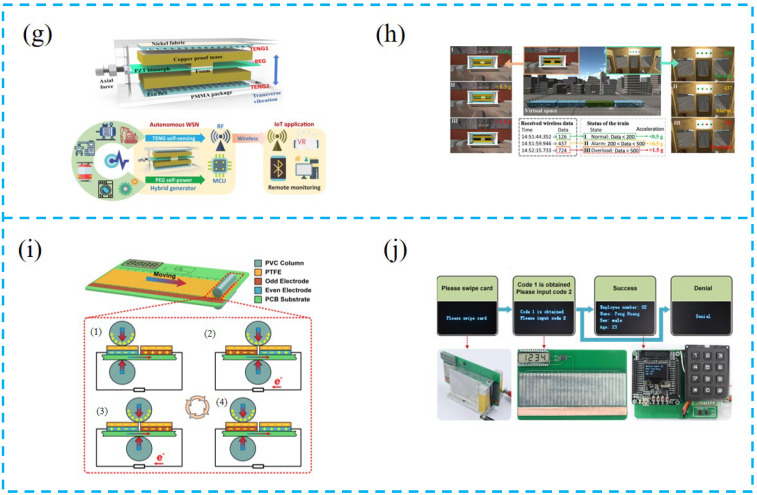
(**a**) Photographs of 140 commercial blue and green LEDs connected in series directly lightened by 2% Ca-BZT-based HNG without any rectification. (**b**) Charging curves of various capacitors with the capacitances of 0.47, 2.2, 4.7, 22, and 47 μF, rectified voltage of 2% Ca-BZT-based HNG device. (**c**) Instantaneous activation of 70 LEDs and 180 LEDs under periodic hand compression for device areas of 1 × 1 cm^2^ and 3 × 3 cm^2^, respectively. (**d**) (**1**) The open-circuit voltage and (**2**) the short-circuit current under a repeatable hand compressive process for a device area of 1 × 1 cm^2^. (**3**) Working electric circuit of the PTNG-based self-charging system. (**4**) Charging a commercial capacitor with the PTNG device. (**e**) (**1**) 60 green LEDs lit up using the PCF-HG device under the compressive force (**2**) and (**3**) demonstration of self-powered electronics using the PCF-HG device by powering a wristwatch and a temperature sensor. (**f**) (**1**) Commercial capacitor charging using individual components such as TENG, PENG, and hybrid combinations. (**2**) Cyclic stability test using a commercial 33 μF capacitor. (**g**) Structure design of hybrid TENG and PEG module, and schematic illustration of autonomous WSN for equipment vibration monitoring. (**h**) VR demonstration of monitoring status of the train, and wireless acceleration data stream received in serial port software on the host computer. (**i**) Schematic diagram of device operation. (**j**) As an application prototype, a piezoelectric-based active code of “2019” and a triboelectric-based self-driven code of “1234” were synthesized by logical operation “or”, and the obtained final secret code was used for personal identification.

The PTCNG can be combined with an external circuit to continuously supply power for electronic equipment, or it can be combined with a capacitor to form a battery power unit to form a self-powered system. Patnam et al. converted the AC signal output by the hybrid device into a DC signal through the bridge rectifier circuit (Figure 10a,b). To verify the device performance, the capacity values are 0.47, 2.2, 4.7, 22, and 47 μF commercial capacitor charges, and the instantaneous output can light 140 LED lights, which can be used as a power supply for self-powered electronic devices [121].

He et al. prepared a 3 × 3 cm^2^ PTCNG with an output current capable of lighting 180 commercial LEDs (Figure 10c,d), with a high piezoelectric output due to the enhanced surface piezoelectricity of ZnO NFs, and with a high triboelectric output voltage due to the significant enhancement in electrification of the Au–PDMS contact, which enables this device not only to collect human activity and its small mechanical energy, but also to serve as a self-powered charging power source [122].

Vivekananthan et al. prepared a dual-chalcogenide PTCNG (Figure 10e,f). To verify that the device has the ability to charge and discharge, it was tested with power capacitors, LEDs, and watches, and this device can charge capacitors to 2.5 V in 25 s. Furthermore, the device has good stability over the entire cycle of 1500 s, and can instantaneously light up 60 LEDs, as well as 100 μF to power watches. This work provides an opportunity for future development of high-output, stable, built-in self-powered devices [123].

Wang et al. proposed a set of autonomous wireless sensor networks using PTCNG (Figure 10g,h). PENG and TENG impedance matching was used to optimize the broadband performance and tunable resonant frequency of PTCNG to achieve good sensing performance, which can light up 30 series LEDs for sinusoidal vibration and 20 series LEDs for shock vibration. The self-powered accelerometer has a sensitivity of 15 V/g (0–1.5 g) with an optimized 1.5 mm separation gap and good linearity, in addition to continuously powering the Arduinonano and RF transceiver, and sending acceleration signals via a wireless module in combination with artificial intelligence to realize a virtual reality train monitoring demonstration, extending the prospects of WSN for a wide range of future applications [124].

PTCNG can also be applied to identification technology. Wen et al. proposed an intelligent identification system with high security (Figure 10i,j), a high recognition rate, and self-powered, using PVDF of different lengths arranged in binary order to form a piezoelectric active code. Relative sliding triboelectricity was used to form a self-driven code, and the two coded messages were combined to form a self-powered dual-authentication microsystem (DAM). The results verify that these features can improve the recognition rate up to 98%, the maximum load output power density of RS-TENG is 51.2 μW/cm^2^, and the obtained power can drive the LCD with pre-compiled information (i.e., 1234) for about 26 s [125].

Although PTCNGs have a high-output performance, they can only power low-power electronic devices, such as the use of a multi-layer stacking design to enhance output and other methods, so that they can be applied to different power systems as soon as possible.

## 5. Conclusions and Perspectives

With the increase of the world’s demand for renewable energy, the development of green energy harvesters becomes ever more important. As a result, the design of hybridized generators such as PTCNG units has become a promising research field for energy-harvesting and environmental sensing systems. This review systematically reported the progress of PTCNG technology, in terms of electrode fabrication, structural design, and integration concepts. Applications in energy-harvesting and self-powered sensors, as well as their further integration with other potential technologies, were also introduced from the mechanisms, including the methods of charge generation and energy-boosting. Finally, the outlooks and conclusions about future development trends of PTCNG technologies are discussed toward multifunctional and intelligent systems.

Overall, PTCNG is aiming to satisfy the energy and sensing needs of a range of electronic devices and adapt to a variety of working environments. These studies demonstrate new approaches to developing hybrid techniques and novel assemblies for efficiently harvesting environmental energy from a number of sources.

However, there are still some problems that need to be solved, which not only limit the output performance, but also limit their possible future applications:

1. Although the output of PTCNG is higher than that of single nanogenerators, as an important component of self-powered systems, it is far from enough to power some larger power electronic products. In the future, we can enhance the research on surface modification of functional layers, such as physical modification and molecular engineering, to change the triboelectric layer morphology, chemical modification, or add piezoelectric materials and high dielectric constant materials in a polymer or organic matrix to improve the overall device performance output.

2. Nowadays, there are also many different structural designs for PTCNG, but in general, most of the research only makes a simple stacking design for all layers of the device, which limits a wider range of use of the device and fails in simplification. Multilayer structures not only waste the use of materials, but also easily cause system errors and deteriorate the reliability, leading to device performance degradation. It is hoped that future research will explore from the perspective of functional layers, and a smaller number of layers can meet the requirements of all functions and achieve the simpler design via structural integration and miniaturization.

3. While pursuing higher output performance, the trade-off between durability and stability of the device may be neglected, and these two properties determine whether the future device can be used in practice. The triboelectric layer material, especially the triboelectric layer surface, is designed to increase the triboelectric microstructure. However, in long-term use, such designs are more likely to cause damage and wear, hence the need to study the triboelectric materials with self-healing or low-wear properties. In addition, in most of the research on monitoring self-powered systems, the whole system can only run for a short period of time, and it is necessary to improve the stability of the device.

4. With the rapid development of artificial intelligence, many industries are involved in some of its applications. For PTCNG, most of the research simply integrates the device with other functional sensors to make a self-powered monitoring system and then proves that the system has certain effects through simulation experiments. This simple integration does not reach the practical application conditions of future devices. In this era of the Internet of Things, we need to fully combine the two to enable intelligent monitoring integration for a wider range of applications in the future, such as self-powered wearable devices, medical devices, environmental monitoring, and large-scale energy harvesting, with higher reliability, a simpler structure, and lower power consumption.

## Data Availability

Not applicable.
